# Physiological and metabolic analyses provide insight into soybean seed resistance to *fusarium fujikuroi* causing seed decay

**DOI:** 10.3389/fpls.2022.993519

**Published:** 2022-10-20

**Authors:** Xiaoli Chang, Xinyuan Li, Hongbai Meng, Hongju Li, Xiaoling Wu, Guoshu Gong, Huabao Chen, Chunping Yang, Min Zhang, Taiguo Liu, Wanquan Chen, Wenyu Yang

**Affiliations:** ^1^ College of Agronomy & Sichuan Engineering Research Center for Crop Strip Intercropping system, Sichuan Agricultural University, Chengdu, China; ^2^ State Key Laboratory for Biology of Plant Diseases and Insect Pests, Institute of Plant Protection, Chinese Academy of Agricultural Sciences, Beijing, China

**Keywords:** soybean, seed decay, *F. fujikuroi*, metabolites, metabolic pathway, isoflavone biosynthesis

## Abstract

Seed-borne pathogens cause diverse diseases at the growth, pre- and post-harvest stage of soybean resulting in a large reduction in yield and quality. The physiological and metabolic aspects of seeds are closely related to their defense against pathogens. Recently, *Fusarium fujikuroi* has been identified as the dominant seed-borne fungi of soybean seed decay, but little information on the responses of soybean seeds induced by *F. fujikuroi* is available. In this study, a time-course symptom development of seed decay was observed after *F. fujikuroi* inoculation through spore suspension soaking. The germination rate and the contents of soluble sugar and soluble protein were significantly altered over time. Both chitinase and β-1,3-glucanase as important fungal cell wall–degrading enzymes of soybean seeds were also rapidly and transiently activated upon the early infection of *F. fujikuroi*. Metabolic profile analysis showed that the metabolites in glycine, serine, and threonine metabolism and tryptophan metabolism were clearly induced by *F. fujikuroi*, but different metabolites were mostly enriched in isoflavone biosynthesis, flavone biosynthesis, and galactose pathways. Interestingly, glycitein and glycitin were dramatically upregulated while daidzein, genistein, genistin, and daidzin were largely downregulated. These results indicate a combination of physiological responses, cell wall–related defense, and the complicated metabolites of soybean seeds contributes to soybean seed resistance against *F. fujikuroi*, which are useful for soybean resistance breeding.

## Introduction

Soybean is one of the important oilseed crops and rich in high-quality vegetable protein, but soybean seed quality and yield are often affected by seed-borne diseases (Wrather et al., 2003). These diseases frequently occur and severely decrease seed quality and reduce seed germination and vigor. Some infected seeds can even act as important carriers to spread important diseases over long distances (Lv and Sun, 2007). Seed decay is one of the most damaging seed-borne diseases at the pre- and post-harvest stage of soybean. In 1985, *Phomopsis longicola* Hobbs was firstly reported to cause seed decay (Hobbs et al., 1985), and during 1996–2007, this pathogen resulted in soybean yield loss as high as 4.5 million tons in North America (Wrather and Koenning, 2009). Several species in the *Phomopsis/Diaporthe* species complex such as *Diaporthe phaseolorum* var. *caulivora*, *D. phaseolorum* var. *meridionalis*, and *D. phaseolorum* var. *sojae* are also the pathogens of soybean seed decay as well as pod blight (Zhang et al., 1998; Li et al., 2010; Petrović et al., 2016). Chiotta et al. (2016) found that *Fusarium graminearum* and *F. meridionale* were able to infect soybean leading to pod blight and seed decay. More recently, Chang et al. (2020a) reported that *F. fujikuroi*, *F. proliferatum*, *F. verticillioides*, *F. asiaticum*, and *F. incarnatum* were the seed-borne pathogens of soybean under the maize–soybean intercropping in Southwest China. Within these *Fusarium*, *F. fujikuroi* [teleomorph *Gibberella fujikuroi*], one member of the polyphyletic taxon in the *G. fujikuroi* species complex (GFSC), is previously well known as the seed-borne pathogen of rice bakanae diseases (Sun and Snyder, 1981; O'Donnell et al., 1998; Lesile et al., 2005; Summerell et al., 2010) but was later found causing devastating diseases in other economically important plants including sugarcane, wheat, and maize (Cen et al., 2020). In addition, *F. fujikuroi* produces a variety of secondary metabolites such as fumonisins and gibberellins, which result in serious economic losses and threaten human and livestock health (Suga et al., 2019). To effectively reduce the seed damage caused by *F. fujikuroi*, it is therefore necessary to elucidate the seed infection mechanism of *F. fujikuroi* as well as the seed resistance mechanism of its hosts.

During the interaction of pathogens and plants, the preformed physical barriers as well as a range of induced resistance in plant hosts are the important defense lines to defend against pathogens (Dodds and Rathjen, 2010). Seed coat is the key physical barrier of plant seeds to confront diverse pathogens surrounding them, and this layer of resistance not only depends on its structure such as the integrity, thickness, fissures, and cavities of the fenestrated tissue cells but is also closely correlated with diverse chemical compounds produced by the seed coat (Soni et al., 2020). Liang et al. (2000) reported that peanut varieties resistant to *Aspergillus flavus* were characterized by thicker cell walls, more tightly bound epidermal cells, and denser fenestra layer than disease-susceptible varieties. Once the preformer barriers fail to prevent pathogens, a range of induced resistance are sequentially activated, typically through the enhancement of plant cell walls through callose deposition at infection sites (Chen and Kim, 2009; Luna et al., 2011), the production of reactive oxygen species (ROS) as antifungal and signaling molecules (Torres et al., 2006), and the secretion of other antifungal compounds (González-Lamothe et al., 2009). In addition, some pathogenesis-related (PR) proteins such as chitinases and β-1,3-glucanases can be induced to directly target the fungal cell wall structure (Yeoh et al., 2012; Mao et al., 2014), inhibit fungal mycelial growth, and reduce the spore germination of pathogenic fungi (Maher et al., 1993; Egea et al., 1996).

Metabolites often are closely related to seed defense against pathogens (Deng et al., 2017; Li et al., 2021). The phenylpropane pathway is the most important metabolic pathway, and it can upregulate the biosynthesis of secondary metabolites flavonoids, isoflavones, and phenolics, which have been proven as phytoalexins in response to biotic and abiotic stimuli (Steele et al., 1999; Jung et al., 2000). The biosynthesis of these metabolites can be regulated by several genes including flavanone 3-hydroxylase (*F3H*), isoflavone synthase (*IFS*), chalcone synthase (*CHS*), chalcone isomerase (*CHI*), dihydroflavonol 4-reductase (*DFR*), isoflavone reductase (*IFR*), and anthocyanidin synthase (*ANS*) (Chen et al., 2019). In addition, some small-molecule metabolites such as terpenoids and alkaloids are rapidly accumulated in large quantities to enhance plant resistance. Lignin, as an important component of the secondary wall, maintains the basic life process of living organisms (Miedes et al., 2014). Deng et al. (2017) reported that the biosynthesis and accumulation of alkaloids in the seed coat of resistant soybean germplasm provide high antifungal activity to preharvest field mold caused by the infection of *F. verticillioides.* Although metabolomics, as an indispensable link between genes, proteins, and phenotypes, has widely been used in the study on plant chemical defense mechanism in the plant–pathogen interactions (Shi et al., 2021), the information on the key defense-related metabolites and corresponding metabolic pathways of soybean seeds in response to seed-borne pathogens is still far uncovered.

In the present study, we tested the physiological and metabolic responses of soybean seed induced by *F. fujikuroi* after spore suspension soaking at varied time intervals; in particular, the key metabolites and metabolic pathways responsible for seed resistance were explored through metabonomic analysis. It is predicted that this study will provide a useful reference for the better understanding of the seed resistance mechanism and for soybean resistance breeding against seed-borne *F. fujikuroi*.

## Materials and methods

### Pathogen inoculation and disease assessment

*F. fujikuroi* (isolates No. S88) was previously isolated from soybean rotted seeds in Zigong, Sichuan, China, and was identified based on *translation elongation factor 1 alpha* (*EF-1α*) and *DNA-directed RNA polymerase II second largest subunit* (*RPB2*) genes (Chang et al., 2020a). This fungi was cultured on potato dextrose agar (PDA, 200 g·l^-1^ potato, 15 g·l^−1^ agar and 10 g·l^−1^ glucose anhydrous) containing 50 μg·ml^-1^ streptomycin at 25°C for 3 days. For sporulation, a mung bean liquid medium was prepared by boiling 30 g of mung bean in 1 liter of sterilized water for 20 min, filtering the mixture with cheesecloth, and then autoclaving at 121°C for 30 min (Zhou et al., 2016). Five mycelial plugs of *F. fujikuroi* (5 mm in diameter) were transferred into 30 ml of the mung bean liquid medium in an Erlenmeyer flask and shaken at 25°C, 150 r·min^-1^ for 4 days to obtain the spore suspension with a concentration of 1 × 10^5^ spores per milliliter.

The soybean seeds of the cultivar Nandou 12, moderately susceptible to *F. fujikuroi*, were rinsed with running tap water for 20 min, surface-disinfected with 1% NaClO for 3 min, and washed with sterile water three times. After that, the remaining water of the seed surface was finally dried with sterile filter paper. Disinfected seeds were soaked into the spore suspension of *F. fujikuroi* for 5 min and placed on petri plates containing PDA medium. The seeds soaked in a mung bean soup medium were used as the inoculated controls at 0 day post inoculation, while those seeds soaked in sterilized water for 0–7 days were designed as negative controls as compared to the corresponding inoculated seeds. Treatments at each time points contained 5 plates with 10 seeds on each one, and three independent experiment replicates were conducted. All petri plates were incubated at 25°C in the dark in a constant temperature incubator.

From 0 to 7 days post inoculation of *F. fujikuroi*, the mycelial coverage area on the seed surface and the rot symptoms inside the seeds were observed. The germination rate, mycelial coverage percentage (MCP), and disease severity index (DSI) were calculated day by day as described by Chang et al. (2020a). The disease grade was evaluated with the range of 0–4 grade as follows: 0 = healthy seed germination without discoloration inside the seeds; 1 = delayed germination with negligible or no discoloration inside the seeds; 2 = low germination with slightly water-soaked and yellow symptoms inside the seeds; 3 = no germination with partially water-soaked, yellow or brown, softened decay inside the seeds; and 4 = no germination, brown, and severe seed decay. The DSI was calculated according to the formula as follows:


$$\text{DSI=}\frac{\sum \text{(severity grade x seeds per grade)}}{\text{total seeds x the highest severity grade}}x100$$

### Determination of the contents of soluble protein and soluble sugar

Soybean seeds were collected from 0 to 7 days post inoculation of *F. fujikuroi* and used to analyze the contents of soluble protein and soluble sugar. A total of 0.5 g of soybean seeds were rapidly ground with liquid nitrogen into powder and were then transferred into 7 ml of extract solution (1% PVP, 2 mM pH 8.0 EDTA, 0.04% β-mercaptoethanol) in a 10-ml Eppendorf centrifuge tube. After ice bath for 30 min, the extract was centrifugated at 4 000 r·min^-1^ for 20 min, and then, the supernatant was collected into a tube with a volume scale. The soluble protein content of the supernatants was determined through the Coomassie brilliant blue method as described by Xiong (2003).

The soluble sugar content of soybean seeds was examined using the anthrone colorimetry method (Li, 2002). A total of 0.5 g of well-ground seed powder was homogenated with distilled water to a final volume of 25 ml, and then, 10 ml of the homogenate solution was centrifugated at 4000 r·min^-1^, 4°C for 3 min. The supernatants were used as the crude enzyme solution and were gradient-diluted with distilled water. The diluted enzyme solution was fully mixed with 2.5 ml of 0.2% anthrone solution, and then the OD_630_ value of the mixture was examined at room temperature using the equal volume of distilled water as a control. Three replicates were tested for each sample. The contents of soluble sugar were calculated by a standard curve method that was prepared based on a standard sucrose solution.

For both soluble sugar and soluble protein, five plates were prepared for each inoculated time points from 0 to 7 dpi, and 10 seeds were placed on each plate. There were three independent replicates.

### Activity assay of chitinase and beta-1,3-glucanase

The chitinase activity of soybean seeds after *F. fujikuroi* inoculation was examined according to Wu et al. (2007). Approximately 0.5 g of well-ground seed powder of soybean was mixed gently with 2 ml of acetic acid extract (0.05 M, pH 5.0), centrifuged at 12000 r·min^-1^, 4°C for 15 min, and then the supernatants were stored at 4°C as the crude extract enzyme of chitinase. The reaction solution was prepared with 1.5 ml of colloidal chitin solution, 0.5 ml of 0.1 M acetate buffer (pH 4.5), 0.4 ml of enzyme solution, and 0.1 ml of 75 μM sodium azide solution and was then incubated at 37°C for 2–4 h followed by supplementing with 0.5 ml of 0.8 M sodium borate buffer (pH 9.1). After centrifugation at 4000 r·min^-1^ for 5 min, the supernatants of the reaction solution were collected, and approximately 1.5 ml volume were mixed with chitin exonuclease to produce N-acetylglucosamine as treatments. The same volume of the reaction solution was used as a standard control. Finally, 2 ml of potassium pertechnetate solution was added to the treatment and a standard control, respectively. After keeping in boiling water at 100°C for 15 min, the optical density was read at 420 nm using distilled water as a blank control. The contents of N-acetylglucosamine were calculated by a standard curve method.

The crude enzyme solution of β-1,3-glucanase was prepared as chitinase above. For reaction solution, kombucha polysaccharides preheated at 50°C for 3 min was mixed with the same volume of crude enzyme dilution when mixed with distilled water as blank controls. The reaction mixture was incubated at 50°C for 1 h, supplemented with 2 ml of the DNS reagent, and then rapidly incubated in boiling water bath for 5 min. The reaction was stopped by adding 12 ml of distilled water, and the OD_540_ values of all reaction samples were measured by zeroing the blank controls. Three replicates were prepared for each sample with at least three independent experiments. A standard curve of glucose solution was prepared as follows:


$$y = 0.001x - 0.0428,{\text{R}}^{2}= 0.9991$$

### Metabolites analysis using Ultra Performance Liquid Chromatography Tandem Mass Spectrometry

For metabolic profile analyses, soybean seeds after *F. fujikroi* through the spore suspension soaking method were collected after 0, 3, and 5 days, immediately frozen in liquid nitrogen, and stored at -80°C for further analysis. Each treatment time contained six biological replicates. Approximately 100 mg of seed samples with six biological replicates each treatment were individually grounded with liquid nitrogen into well power. The homogenate was resuspended with precold 80% methanol and 0.1% formic acid, incubated on ice for 5 min, and centrifuged at 15,000 g, 4°C for 20 min. The supernatant was diluted to final concentration using 53% methanol in LC-MS grade water, subsequently transferred to a new Eppendorf tube, and centrifuged at 15,000 g, 4°C for 20 min. Finally, the obtained supernatant was injected into the liquid chromatography-tandem mass spectrometry (LC-MS/MS) system. The same volume of all tested samples was mixed as quality control (QC) samples to balance the state of the LC-MS/MS system and the detection instrument for the evaluation of the system stability. Meanwhile, the blank samples were set to remove background ions.

Ultra performance liquid chromatography-tandem mass spectrometry (UPLC-MS/MS) analyses were performed using a Vanquish UPLC system (Thermo Fisher, Dreieich, Germany) coupled with an Orbitrap Q Exactive^TM HF^ mass spectrometer (Thermo Fisher, Dreieich, Germany) in Novogene Co., Ltd. (Beijing, China). Samples were injected into a Hypesil Gold column (100 × 2.1 mm, 1.9 μm) using a 17-min linear gradient at a flow rate of 0.2 ml·min^-1^. A Q Exactive™ HF mass spectrometer was operated in both positive and negative polarity mode with a spray voltage of 3.2 kV, a capillary temperature of 320°C, a sheath gas flow rate of 40 arb, and an aux gas flow rate of 10 arb. Eluent A (0.1% formic acid in water) and eluent B (methanol) were used for the positive polarity mode, while eluent A (5 mM ammonium acetate, pH 9.0) and eluent B (methanol) were used for the negative polarity mode. The solvent gradient was set as follows: 2% B for 1.5 min, 2%–100% B for 12.0 min, 100% B for 14.0 min, 100%–2% B for 14.1 min, and 2% B for 17 min.

### Data processing of metabolic profile and metabolite annotation

The raw data files generated by UPLC-MS/MS were processed using the Compound Discoverer 3.1 (CD3.1, Thermo Fisher, Waltham, MA USA). The peak alignment, peak picking, and quantitation for each metabolite were analyzed. The main parameters were set as follows: a retention time tolerance of 0.2 min, an actual mass tolerance of 5 ppm, a signal intensity tolerance of 30%, a signal/noise ratio of 3:1, and a minimum intensity of 100 000. After that, peak intensities were normalized to the total spectral intensity. The normalized data were used to predict the molecular formula based on additive ions, molecular ion peaks, and fragment ions. Metabolite peaks were then matched with the mzCloud (https://www.mzcloud.org/), mzVault, and MassList databases to obtain the accurate qualitative and relative quantitative results. Statistical analyses were conducted using the statistical software R (R version R-3.4.3), Python (Python 2.7.6 version), and CentOS (CentOS release 6.6). Normal transformations were attempted using the area normalization method when data were not normally distributed.

These metabolites were annotated through the Kyoto Encyclopedia of Genes and Genomes (KEGG) database (https://www.genome.jp/kegg/pathway.html). Principal component analysis (PCA) and partial least squares discriminant analysis (PLS-DA) were conducted at metaX. Univariate analysis (t-test) was applied to calculate the statistical significance (P-value). The metabolites with a variable importance in projection (VIP) score > 1 and p-value< 0.05 and fold change (FC) ≥ 2 or FC ≤ 0.5 were considered to be differential metabolites (DMs). Volcano plots were used to filter the metabolites of interest based on the log2 (fold change) and -log10 (p-value) of metabolites. For clustering heat maps, data were normalized using the z-scores of the intensity areas of differential metabolites and were plotted by the Pheatmap package in R language. The correlation between differential metabolites were analyzed by cor () in R language (Pearson index). The statistical significance of the correlation between differential metabolites was calculated by cor.mtest() in R language. The p-value< 0.05 was considered as statistically significant, and correlation plots were plotted by the corrplot package in R language. The functions of these metabolites and metabolic pathways were studied using the KEGG database. For the analysis of the metabolic pathway enrichment of different metabolites, metabolic pathways were considered as enrichment when ratios were satisfied by x/n > y/N, and metabolic pathways were considered as statistically significant enrichment when the p-value of the metabolic pathway< 0.05. For the analysis of different metabolites and metabolic pathways, groups were designed as S88_3 vs S88_0, S88_5 vs S88_3, and S88_5 vs S88_0.

## Results

### Disease symptoms of soybean seeds after *F. fujikuroi* inoculation

**Figure 1:**
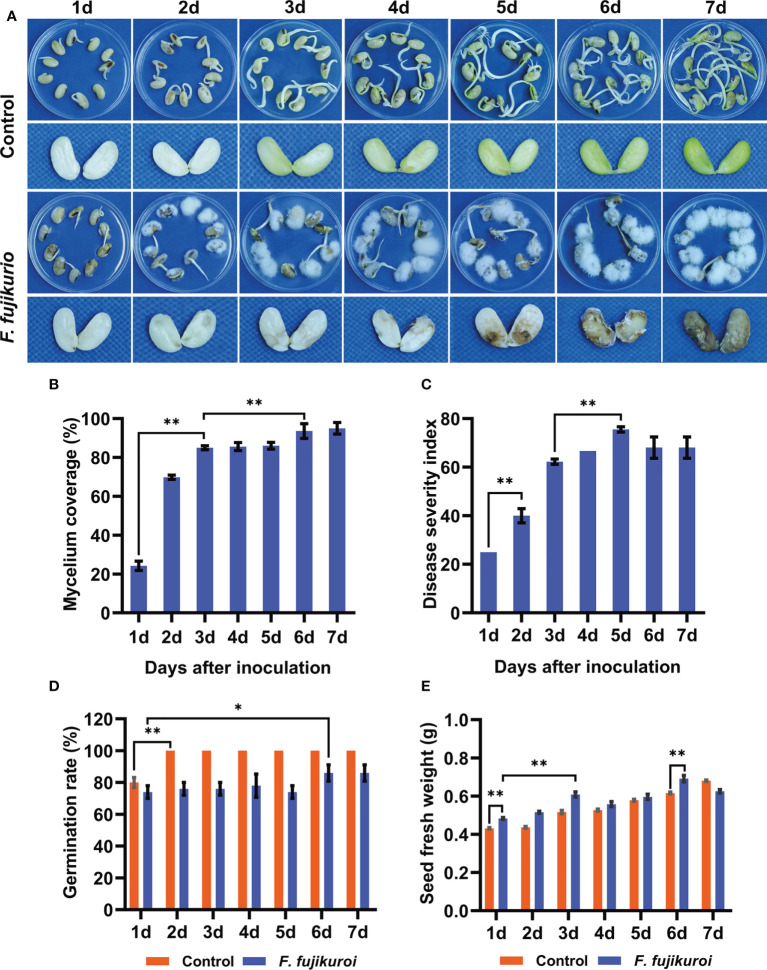
Symptoms of seed rot caused by *F. fujikuroi* and related seed parameters. **(A)**Time-course symptoms of soybean seeds with or without *F. fujikuroi* inoculation were observed over time. **(B)** Percentage of mycelium coverage was calculated by the coverage area of mycelium on the seed surface. **(C)** Disease severity index (DSI) was evaluated and calculated according to disease symptoms. Germination rate **(D)** and seed fresh weight **(E)** of the non-inoculated and inoculated seeds with *F. fujikuroi* were recorded over the inoculation time. Statistical analysis was conducted using Statistical Product and Service Solutions (SPSS) version 23.0 with the ANOVA method; ** indicates significant difference at the level of p = 0.01, while * stands for significant difference at the level of p = 0.05. Each treatment contains five plates with 10 seeds on each plate (n = 50), and three independent experiments were conducted.

To uncover the time-course symptoms of soybean seeds after *F. fujikuroi* infection, the mycelial growth on the seed surface and the rot symptoms inside the seeds were evaluated. We found that the mycelium of *F. fujikuroi* rapidly grew on the seed surface, and typical symptoms were characterized by water-soaked, brown, and even rot inside the seeds over time when compared with non-inoculated control seeds (**Figure 1A**). The percentage of mycelium coverage increased sharply after 3 dpi, shortly remained, and continuously reached up to 93.60% at 6 dpi followed by severe seed rot inside (**Figure 1B**). The DSI had almost the same mycelium coverage but went up the peak at 5 dpi (**Figure 1C**). In addition, the germination of inoculated seeds was significantly suppressed (p<0.01) and only reached 86% at 7 dpi, whereas the non-inoculated seeds totally germinated at 2 dpi (**Figure 1D**). Upon *F. fujikuroi* infection, the seed fresh weight significantly increased over the inoculation time and peaked at 6 dpi because of abundant mycelium growing on the seed surface (p<0.05), and they were much higher than those non-inoculated seeds (**Figure 1E**).

### Physicochemical parameters of soybean seeds infected by *F. fujikuroi*

**Figure 2:**
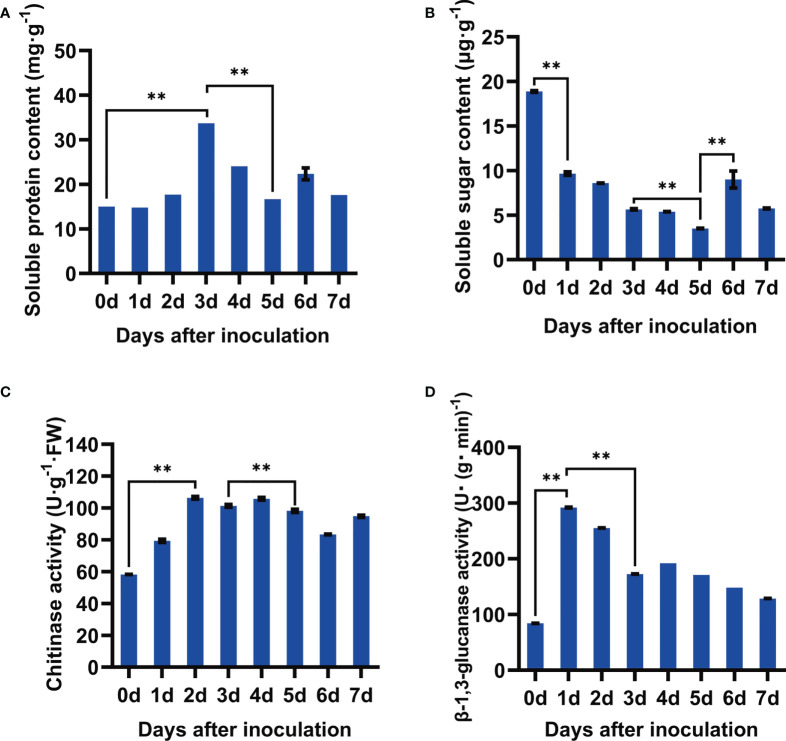
Determination of physiological and chemical characteristics of soybean seeds after inoculation with *F. fujikuroi*. **(A)** Soluble protein content. **(B)** Soluble sugar content. **(C)** Activity assay of chitinase. **(D)** Activity assay of β-1,3-glucan. Each treatment contains five plates with 10 seeds on each plate (n = 50), and three independent experiments were conducted. Statistical analysis was conducted using SPSS with the ANOVA method, and ** indicates significant difference at the level of p=0.01.

As the important seed responses to pathogen infection, soluble protein and soluble sugar were examined after *F. fujikuroi* infection. It showed that the content of soluble protein gradually increased upon pathogen inoculation, peaked at 3 dpi with the content of 33.71 mg·g^-1^, and then significantly went down at 5 dpi followed by severe seed rot (**Figure 2A**). Compared with that at 0 dpi, *F. fujikuroi* infection also caused a rapid decline in the content of soluble sugar until 5 dpi and then slightly increased at 6 dpi in the range of 3.50–18.88 μg·g^-1^ (**Figure 2B**). These results showed that the seeds of Nandou 12 produced more soluble protein to defend against pathogens while a significant decrease in the soluble sugar content contributed to the enhanced respiration of soybean.

Chitinase and β-1,3-glucanase are the important cell wall–degrading enzymes targeting chitin and β-1,3-glucan of the fungal cell wall components, respectively. As shown **Figures 2C, D**, *F. fujikuroi* rapidly activated these two enzymes. Compared to those at 0 dpi, the activities of chitinase in the inoculated seeds rapidly rose up to 106.40 U·g^-1^ FW at 2 dpi and then kept a slight shift in the range of 58.21–106.40 U·g^-1^FW. Similarly, β-1,3-glucanase activity dramatically increased 3.0-fold as compared to that at 0 dpi and then gradually decreased over the inoculation time. Thus, the activation of the cell wall–degrading enzymes chitinase and β-1,3-glucanase might contribute for the early defense of soybean seeds against *F. fujikuroi* infection.

### Qualification evaluation of metabolic data and seed metabolites annotated in response to *F. fujikuroi* inoculation

**Figure 3:**
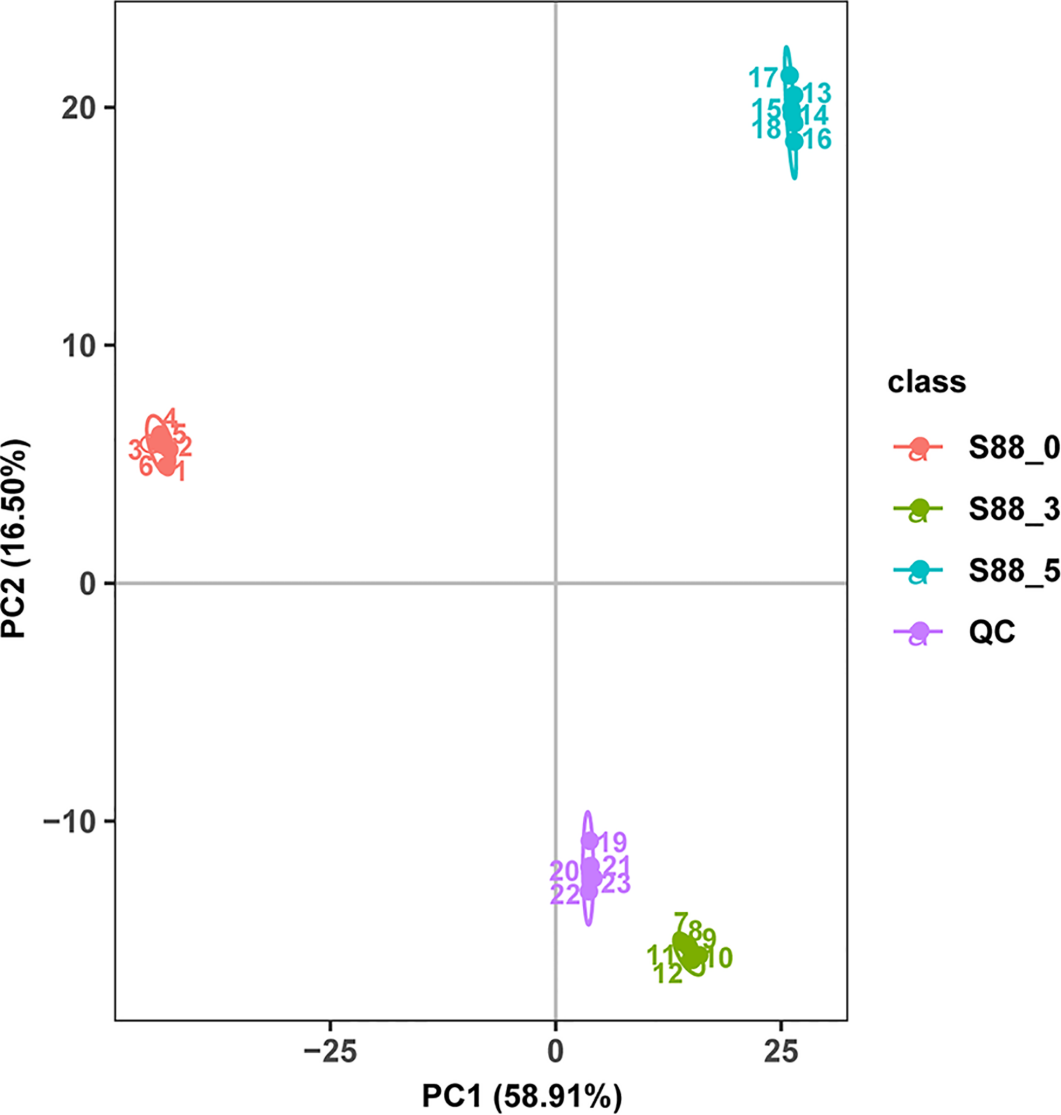
PCA score of different metabolites and the correlation analysis of QC samples. The three samples under three inoculation time points of *F. fujikuroi* were named as S88_ 0, S88_ 3, and S88_ 5, respectively. The six biological replicates corresponding to each sample were named S88_ 0_ a, S88_ 3_ a, and S88_ 5_ a, respectively, where a ∈ {1, 2, 3, 4, 5, 6}.

According to PCA, metabolic profiles at three time intervals postinoculation of *F. fujikuroi* were clearly distributed into three separate groups, in which the first two principal components explained 75.41% of the variability, containing 58.91% [principal component 1 (PC1)] and 16.50% [principal component 2 (PC2)], respectively (**Figure 3**). All six biological replicates at each time interval were clustered together, indicating that little difference exists among biological replicates and the quality of data are very high for further metabolic profile analyses.

**Figure 4:**
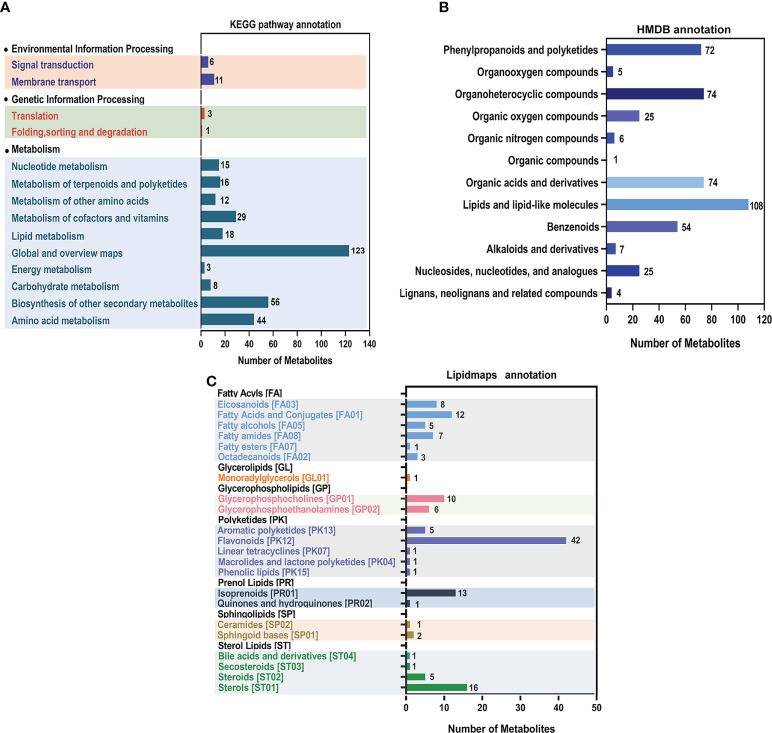
Annotation of metabolites using the KEGG pathway, HMDB categories, and LIPID MAPS. The horizontal coordinates represent the number of metabolites in all three figures. **(A)** The vertical axis represents the annotated KEGG pathways, and the graph shows the number of metabolites annotated in each secondary classification under the pathway primary classification. **(B)** The vertical axis represents the annotated HMDB categories, and the graph shows the number of metabolites annotated to the secondary categories (Super Class) in the HMDB. **(C)** The vertical coordinate represents the annotated LIPID MAPS lipid categories, and the graph shows the number of (lipid) metabolites annotated to the main hierarchical category (Main_Class) under the seven major lipid categories (Categories) in LIPID MAPS.

According to KEGG pathway annotation, the seed metabolites of soybean induced by *F. fujikuroi* were the most highly enriched in the global and overview maps. There were more metabolites enriched in the biosynthesis of other secondary metabolites, amino acid metabolism, carbohydrate metabolism, the metabolism of cofactors and vitamins, and lipid metabolism than other metabolites (**Figure 4A**). Annotation results from the Human Metabolome Database (HMDB) database showed that lipids and lipid-like molecules were the most enriched metabolites of soybean seeds when infected by *F. fujikuroi*, and other compounds such as organic acids and derivatives, phenylpropanoids and polyketides, organoheterocyclic compounds, benzenoids, and organic oxygen compounds were also largely induced by *F. fujikuroi* (**Figure 4B**). Furthermore, the annotation using LIPID MAPS showed that 42 metabolites in the flavonoid pathway were mostly enriched when sterols, isoprenoids, fatty acids, and conjugates also had relatively high accumulation as compared to other metabolites (**Figure 4C**).

### Quantitative analysis of different metabolites of soybean seeds induced by *F. fujikuroi*

**Figure 5:**
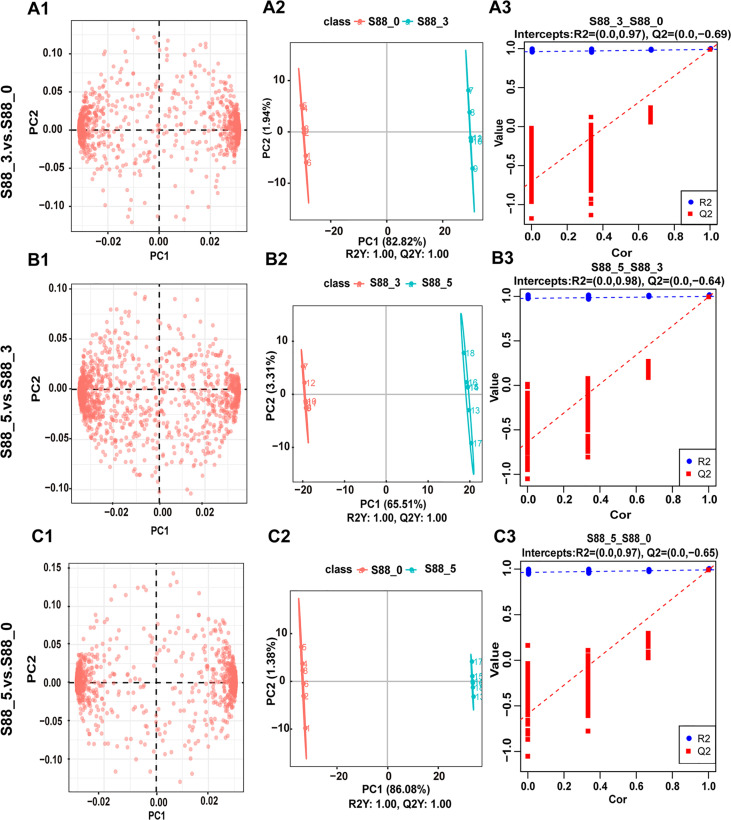
Statistical analysis for metabolite differences among three comparison groups after *F. fujikuroi infection*. Metabolic data of soybean seeds induced by *F. fujikuroi* were statistically analyzed among three comparison groups including S88_3.vs.S88_0, S88_5.vs.S88_3, and S88_5.vs.S88_0. **(A1–A3)** PCA loading plots. **(B1–B3)** PLS-DA score plots. **(C1–C3)** PLS-DA valid plots.

To uncover the key seed metabolites responding to *F. fujikuroi*, different metabolites were analyzed among three comparison groups of S88_3 vs S88_0, S88_5 vs S88_3, and S88_5 vs S88_0 over inoculation time. PCA loading plots showed a significant change in different metabolites in each comparison group (**Figures 5A1, B1, C1**), among which the S88_3.vs.S88_5 group displayed the most diffuse distribution (**Figure 5B1**). For the PLS-DA analysis, three comparison groups were scored 82.82%, 65.51%, and 86.08% in PC1, respectively **(** **Figures 5A2, B2, C2**). After after sevenfold cross-validation, the R^2^ and Q^2^ values in each comparison group were all close to 1 (**Figures 5A3, B3, C3**), implying that the predictive ability and quality of the three groups of models are suitable for subsequent experiments. Thus, based on the PCA and PLS-DA analysis, *F. fujikuroi* induced the significant time-course changes in the metabolites of soybean seeds.

**Figure 6:**
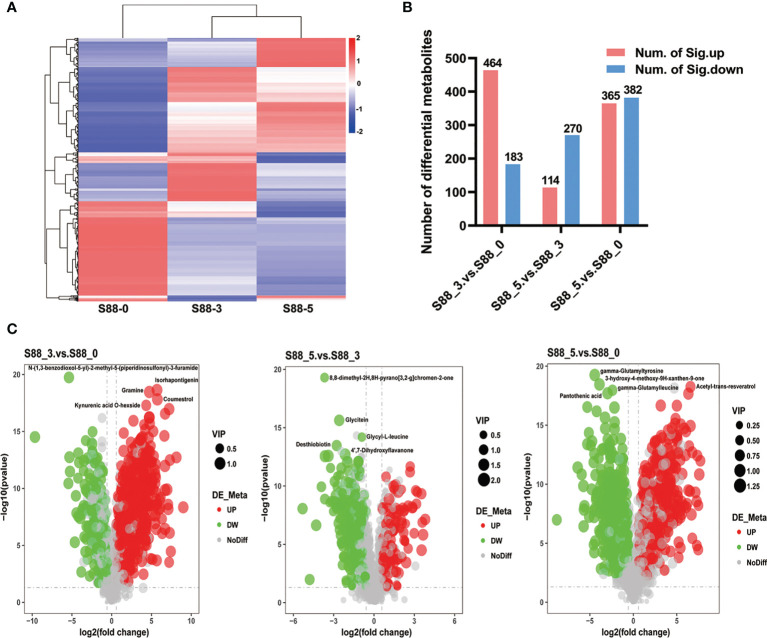
Quantification of different metabolites of soybean seeds after *F. fujikuroi* inoculation over time. **(A)** Longitudinal is the clustering of samples, and cross-sectional is the clustering of metabolites, with shorter clustering branches representing higher similarity. The cross-sectional comparison shows the relationship of the clustering of the metabolite content between groups. **(B)** The graphs indicate the total numbers of significant different metabolites at the corresponding comparison groups, in which red and blue bars stand for the numbers of significantly upregulated (num. of Sig. down) and significantly downregulated different metabolites (num. of Sig. down), respectively. **(C)** Volcano of differential metabolites; the x-axis represents the log_2_ fold change of metabolites in different groups, and the y-axis represents the difference significance level (-log_10,_ P-value). Each dot in the volcanic map represents one metabolite; red, green, and gray dots stand for the significantly upregulated metabolites, significantly downregulated metabolites, and no different metabolites, respectively. The dot size represents the VIP value.

It is clearly seen that *F. fujikuroi* infection remarkably induced the different patterns of upregulated and downregulated metabolites among three comparison groups (**Figure 6A**). There were the most upregulated metabolites such as coumestrol, kynurenic acid-Ο-hexside, gramine, and isorhapontigenin in the S88_3.vs.S88_0 group, when more downregulated metabolites including desthiobiotin, glycitein, glycyl-L-leucine, and 8,8-dimethyl-2H,8H-pyrano [3,2-g] chromen-2-one, 4’7-dihydroxyflavanone, were accumulated rather than upregulated ones in the S88_5.vs.S88_3 group (**Figures 6B, C**). In the S88_5.vs.S88_0 group, the top five different metabolites included pantothenic acid, gamma-glutamylleucine, 3-hydroxy-4-methoxy-9H-xanthen-9-one, gamma-glutamyltyrosine, and acetyl-trans-resveratrol (**Figure 6C**), but there was no significant difference in the numbers of upregulated and downregulated metabolites (**Figure 6B**).

### Different KEGG pathways of soybean seeds activated by *F. fujikuroi* over time

**Figure 7:**
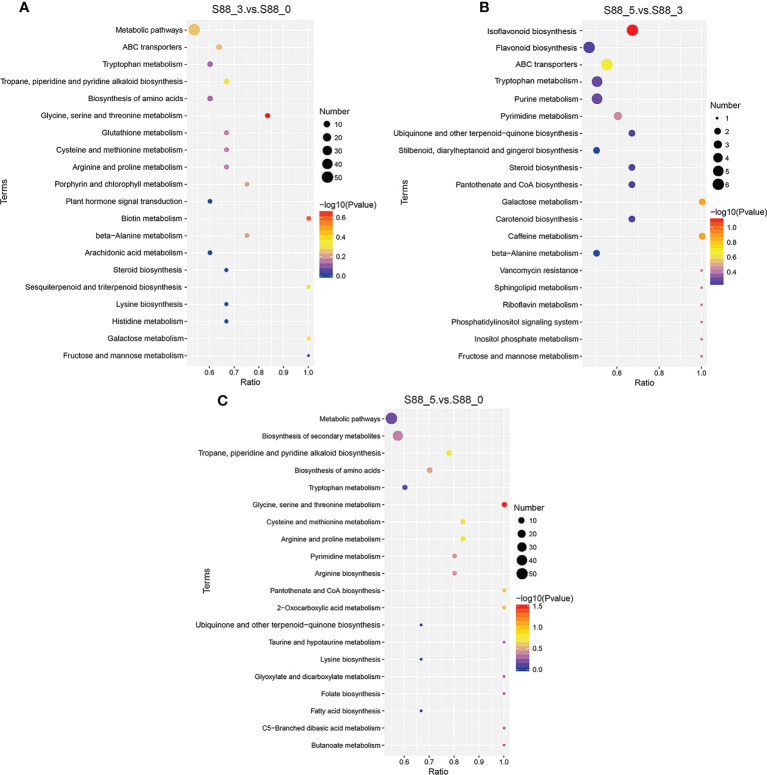
Top 20 KEGG pathways enriched in different comparison groups over the *F. fujikuroi* inoculation time. **(A**–**C)** are the scatter plots of the enrichment statistics of the KEGG pathway in S88_3.vs.S88_0, S88_5.vs.S88_3, and S88_5.vs.S88_0, respectively. The abscissa in the figure is x/y (the rate of the number of differential metabolites in the corresponding metabolic pathway to the total number of metabolites identified in the pathway), and the larger the values indicate, the higher the enrichment of differential metabolites in the pathway. The color of the dot represents the p-value of the hypergeometric test, and the smaller the values stand for, the greater the reliability of the test and the more statistically significant. The size of the dot represents the number of different metabolites in the corresponding pathway. The larger the dot indicates the more differential metabolites in the pathway.

To further uncover the metabolic pathways participating in soybean seed defense against *F. fujikuroi*, the KEGG annotation of seed metabolites was analyzed (**Supplementary Table S1**). In the S88_3.vs.S88_0 group, we found that there are 143 differential metabolites enriched in 35 metabolic pathways. Among them, glycine, serine, and threonine metabolism with 5 different metabolites including L-homoserine, betaine aldehyde, L-tryptophan, L-cystathionine, and betaine, was the most enriched metabolic pathway in response to *F. fujikuroi* infection (**Figure 7A**). In addition, *F. fujikuroi* also activated other metabolic pathways such as biotin metabolism, tropane, piperidine and pyridine alkaloid biosynthesis, porphyrin and chlorophyll metabolism, cysteine and methionine metabolism, and arginine and proline metabolism. In the S88_5.vs.S88_3 group, 65 differential metabolites were enriched in 28 metabolic pathways, and the most relevant metabolic pathway to *F. fujikuroi* infection was isoflavonoid biosynthesis (identified metabolites: formononetin, daidzein, glycitin, glycitein, biochanin A, and genistein), galactose metabolism (identified metabolites: inositol and d-sorbitol), and caffeine metabolism (identified metabolites: 1-methyluric acid and xanthine) (**Figure 7B**). Specifically, the five pathways of galactose metabolism, ABC transporters, fructose and mannose metabolism, tryptophan metabolism and beta-alanine metabolism were overlapped between the S88_3.vs.S88_0 group and the S88_5.vs.S88_3 group. In the S88_5.vs.S88_0 group, a total of 195 differential metabolites were enriched in 39 metabolic pathways; the most relevant metabolic pathway in response to *F. fujikuroi* was glycine, serine, and threonine metabolism involving six metabolites: l-homoserine, betaine aldehyde, l-tryptophan, choline, l-cystathionine, and betaine (**Figure 7C**). Hence, according to the pathway enrichment analysis, flavonoids could be involved in the *F. fujikuroi*-treated seed defense against *F. fujikuroi*.

### Top 20 different metabolites of soybean seeds induced by *F. fujikuroi*

According to the analysis of the key different metabolites of soybean seeds induced by *F. fujikuroi* over time, the top 20 different metabolites in each comparison group were screened based on the p-value and FC (**Table 1**). Our results showed that in the S88_3.vs.S88_0 group, the main different metabolites included isorhapontigenin, coumestrol, 7-hydroxy-3-(4-methoxyphenyl)-4h-chromen-4-one, s-adenosyl-l-homocysteine, myricetin, glycitein, coniferin, and others, and 17 of the top 20 metabolites were upregulated upon *F. fujikuroi* inoculation. In contrast, only columbianetin acetate and nor-9-carboxy-δ9-thc were upregulated in the S88_5.vs.S88_3 group, whereas other metabolites such as glycitein, glycyl-l-leucine, desthiobiotin, 4’,7-dihydroxyflavanone, 6’’-o-acetylglycitin, glycitin, genistein, phloridzin, formononetin, and daidzin, were significantly downregulated. In addition, the different metabolites in the S88_5.vs.S88_0 group mainly included γ-glutamyltyrosine, γ-glutamylleucine, 6-methylquinoline, eriodictyol-7-o-glucoside, dl-tryptophan, l-cystathionine, and menadione. Among them, the different metabolites such as n-(1,3-benzodioxol-5-yl)-2-methyl-5-(piperidinosulfonyl)-3-furamide, n-(4-chlorophenethyl)-n’-(4-chlorophenyl)urea, coumestrol and kynurenic acid o-hexside overlapped in the S88_3.vs.S88_0 group and S88_5.vs.S88_0 group. Except for coumestrol which is upregulated in S88_3.vs.S88_0 group, the other three metabolites were also significantly downregulated. Three metabolites including 6-methylquinoline, dl-tryptopha, and γ-glutamyltyrosine were also the overlapping differential metabolites in the S88_5.vs.S88_3 group and S88_5.vs.S88_0 group, and they were all significantly downregulated at the same time (**Table 1**).

**Table 1:** Comparison of different metabolites of soybean samples in three groups based on the inoculation time of *F. fujikuroi*.

Comparison time group	Metabolites	FC	p-value	Up- or Down-regulated
S88_3.vs.S88_0	N-(1,3-benzodioxol-5-yl)-2-methyl-5-(piperidinosulfonyl)-3-furamide	0.02	1.3х10^-20^	down
	Isorhapontigenin	52.0	2.3х10^-19^	up
	Gramine	26.6	3.4х10^-19^	up
	Coumestrol	53.6	1.5х10^-18^	up
	Kynurenic acid O-hexside	20.2	5.1х10^-18^	up
	7-hydroxy-3-(4-methoxyphenyl)-4H-chromen-4-one	149.1	1.1х10^-17^	up
	S-Adenosyl-L-homocysteine	104.8	2.8х10^-17^	up
	Myricetin	26.5	5.8х10^-17^	up
	LysoPC 12:1	26.5	6.2х10^-17^	up
	Glycitein	6.6	1.2х10^-16^	up
	7-(3,4-dihydroxyphenyl)-5-hydroxy-1-(4-hydroxyphenyl)heptan-3-one	27.3	2.0х10^-16^	up
	N-(4-chlorophenethyl)-N’-(4-chlorophenyl)urea	16.6	2.5х10^-16^	up
	3-methyl-5-oxo-5-(4-toluidino)pentanoic acid	18.7	6.8х10^-16^	up
	IAA-Asp	37.1	9.7х10^-16^	up
	2-{4-[(trifluoromethyl)thio]phenoxy}ethanohydrazide	0.2	1.1х10^-15^	down
	L-Glutamic acid	2.9	1.4х10^-15^	up
	Acetyl-trans-resveratrol	98.5	2.5х10^-15^	up
	Ethyl-5-[(2,1,3-benzoxadiazol-4-ylsulfonyl)amino]-2-piperidinobenzoate	0.01	3.1х10^-15^	down
	9-HpOTre	14.6	3.2х10^-15^	up
	Coniferin	24.5	3.6х10^-15^	up
S88_5.vs.S88_3	8,8-dimethyl-2H,8H-pyrano[3,2-g]chromen-2-one	0.08	5.0х10^-20^	down
	Glycitein	0.17	2.3х10^-16^	down
	Desthiobiotin	0.15	3.4х10^-14^	down
	Coumestrol	0.18	1.1х10^-13^	down
	4’,7-Dihydroxyflavanone	0.23	2.0х10^-13^	down
	6’’-O-Acetylglycitin	0.09	2.6х10^-13^	down
	Glycitin	0.08	2.8х10^-13^	down
	6-Methylquinoline	0.52	3.3х10^-13^	down
	gamma-Glutamyltyrosine	0.26	5.8х10^-13^	down
	5,7-dihydroxy-3-(4-hydroxyphenyl)-4H-chromen-4-one	0.45	8.1х10^-13^	down
	Genistein	0.45	8.2х10^-13^	down
	Uric acid	0.25	8.5х10^-13^	down
	columbianetin acetate	6.46	2.1х10^-12^	up
	DL-Tryptophan	0.52	2.8х10^-12^	down
	ABL 127	0.11	2.8х10^-12^	down
	Phloridzin	0.19	3.0х10^-12^	down
	Formononetin	0.26	4.9х10^-12^	down
	Linoleic acid	0.46	5.8х10^-12^	down
	Nor-9-carboxy-δ9-THC	6.41	6.2х10^-12^	up
	Daidzin	0.38	1.0х10^-11^	down
S88_5.vs.S88_0	gamma-Glutamyltyrosine	0.05	5.5х10^-20^	down
	3-hydroxy-4-methoxy-9H-xanthen-9-one	0.07	3.5х10^-19^	down
	Acetyl-trans-resveratrol	95.18	5.5х10^-19^	up
	Isocryptotanshinone	26.12	7.0х10^-19^	up
	Pantothenic acid	0.13	2.0х10^-18^	down
	4-(3,4-dihydro-2H-1,5-benzodioxepin-7-ylamino)-4-oxobutanoic acid	80.68	2.1х10^-18^	up
	5α-Dihydrotestosterone	8.88	2.1х10^-17^	up
	Kynurenic acid O-hexside	32.14	2.2х10^-17^	up
	N-(1,3-benzodioxol-5-yl)-2-methyl-5-(piperidinosulfonyl)-3-furamide	0.03	2.9х10^-17^	down
	6-Methylquinoline	0.22	3.1х10^-17^	down
	4-(2,3-dihydro-1,4-benzodioxin-6-yl)-1,2-diphenylbut-2-ene-1,4-dione	0.05	6.6х10^-17^	down
	Eriodictyol-7-O-glucoside	0.10	2.3х10^-16^	down
	N-butyl-2-methyl-5-(piperidinosulfonyl)-3-furamide	29.03	2.8х10^-16^	up
	DL-Tryptophan	0.22	5.6х10^-16^	down
	L-Cystathionine	27.77	7.3х10^-16^	up
	N-(4-chlorophenethyl)-N’-(4-chlorophenyl)urea	44.01	8.8х10^-16^	up
	2-(3,5-dichlorophenyl)-6-methyl-2,3,4,5-tetrahydropyridazin-3-one	95.58	1.1х10^-15^	up
	L(-)-Carnitine	21.49	1.7х10^-15^	up
	Menadione	0.11	1.8х10^-15^	down
	3-Methoxy prostaglandin F1α	57.52	1.8х10^-15^	up

FC refers to the difference multiple, which is the ratio of the mean values of all biological repeated quantitative values of each metabolite in the comparison group. the P-value is calculated by the t-test, which indicates the difference significance level. VIP refers to the variable projection importance of the first principal component, which indicates the contribution of metabolites to the subgroup. Up indicates upregulation; down indicates downregulation. The thresholds are set as VIP > 1.0, FC > 1.5 or FC< 0.667 and p-value< 0.05, and the different metabolites.

### *F. fujikuroi* activated the isoflavonoid metabolites in soybean seeds

**Figure 8:**
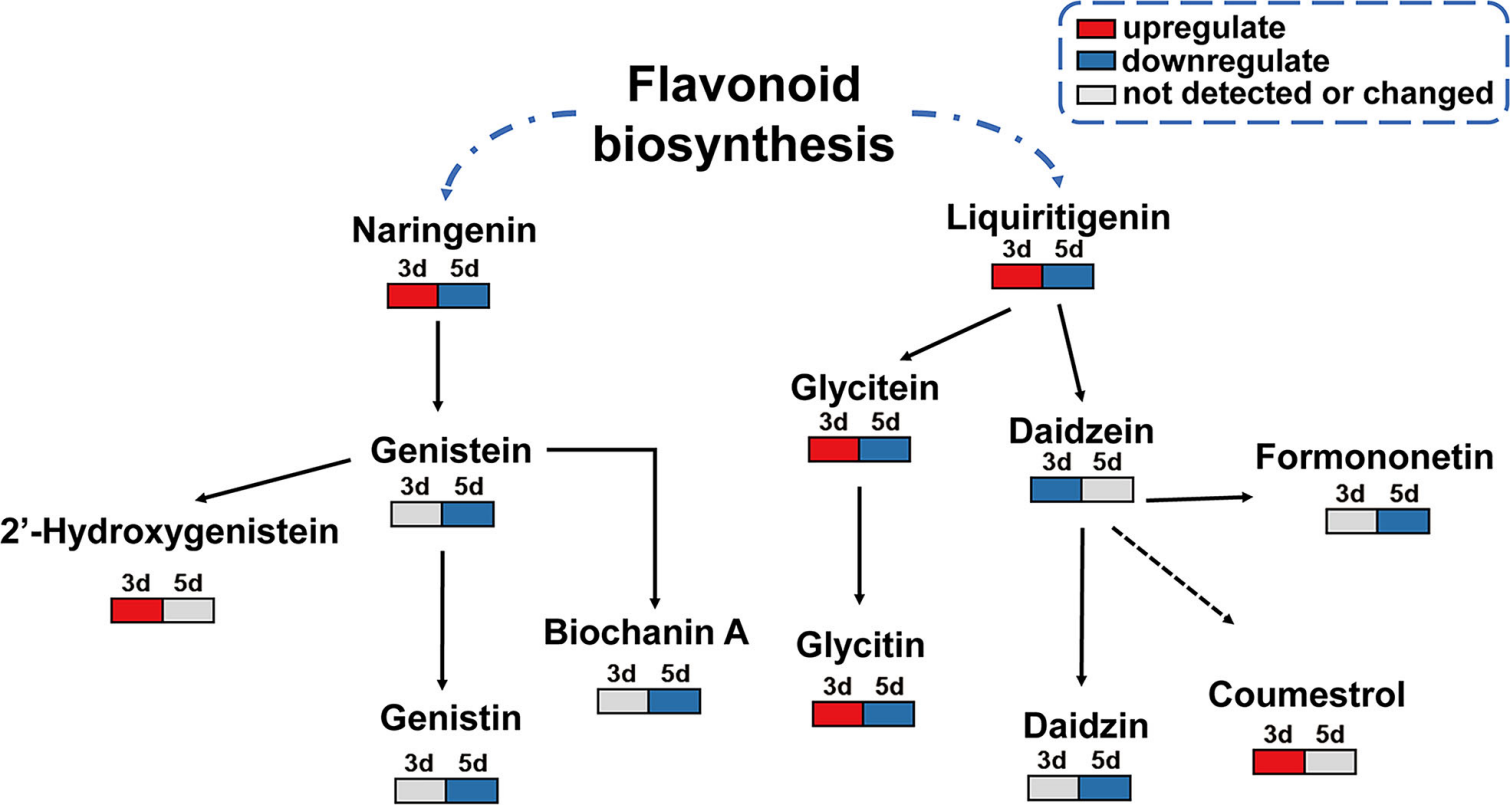
Change model of the key metabolites in the isoflavone biosynthesis pathway. Gray box means undetected or changed metabolites at the indicated time points; the red box indicates the upregulated differential metabolites, and the blue box stands for the downregulated differential metabolites.

Since the isoflavonoid pathway was significantly activated upon *F. fujikuroi* infection, and several metabolites in this pathway showed remarkable changes as the top 20 metabolites, a simple metabolic pathway was drawn to clearly display the change pattern of the key metabolites in this pathway. As shown in **Figure 8**, two upstream metabolites, naringenin and liquiritigenin, were transiently upregulated at 3 dpi and then decreased at 5 dpi. Their downstream metabolites such as genistein, glycitein, and daidzein, showed distinct change patterns. As the downstream of naringenin, genistein was not detectable at 3 dpi but significantly downregulated at 5 dpi. Among three downstream metabolites of genistein, biochanin A and genistin showed the similar change, but 2’-hydroxygenistein was significantly upregulated at 3 dpi. For the downstream metabolites of liquiritigenin, glycitein and its glycitin were significantly and transiently accumulated at 3 dpi and then rapidly decreased, indicating their role in soybean seed resistance to *F. fujikuroi* infection. Additionally, daidzein acts as a central component of the isoflavonoid biosynthetic pathway and the precursor of the soybena phytoalexins glyceollins and coumestrol, and in our results, daidzein was remarkably downregulated at 3 dpi upon *F. fujikuroi* infection and its two downstream metabolites formononetin and daizin were sequentially downregulated at 5 dpi. Moreover, the soybean phytoalexin coumestrol related to daidzein synthesis was rapidly accumulated at 3 dpi, indicating that the accumulation of coumestrol in soybean seeds tissues is the signal of early defense response. Thus, the accumulation of metabolites in the isoflavone biosynthesis pathway contributes to soybean seed resistance to *F. fujikuroi*.

## Discussion

Soybean seed-borne diseases can be caused by several pathogens including the *Phomopsis/Diaporthe* species complex (Hobbs et al., 1985; Petrović et al., 2016), *Fusarium* spp. (Chiotta et al., 2016; Chang et al., 2020a; Chang et al., 2020b) and *Cercospora* spp. (Li et al., 2019) and have been becoming more and more severe with the continuous increase of the soybean planting area and harsh climate at the preharvest period of soybean (Deng et al., 2017; Li et al., 2019). Previously, many efforts have been made to identify the resistance of soybean resources to *Phomopsis* sp. and *Cercospora* sp. (Li et al., 2015; Li et al., 2017; Li et al., 2019), and a range of soybean lines were reported to have certain levels of resistance. However, this study focused on the interaction of soybean and *F. fujikuroi* and on exploring the physiological and metabolic mechanism of soybean seeds to the seed-borne *F. fujikuroi.*

The infection of pathogen often activates a range of the physiological and chemical changes in plant hosts. Seed germination is one of the important evaluation factors of seed quality. Our results showed that *F. fujikuroi* remarkably delayed seed germination until 5 days, which is consistent with previous studies in which inoculation with *P. longicolla* reduced the germination of several soybean lines (Li et al., 2015; Li et al., 2017). In addition, several *Fusarium* spp. were also reported to inhibit seed germination (Purahong et al., 2012; Jiang et al., 2020). Soluble sugars and soluble proteins are the important energy substances for plants, and they can provide energy for not only disease resistance but also the growth and reproduction of the infested pathogens (Morkunas and Ratajczak, 2014; Tarkowski et al., 2019; Formela-Luboińska et al., 2020). In this study, *F. fujikuroi* infection significantly increased the contents of soluble protein at 3 dpi but decreased the contents of soluble sugar, indicating that soybean seeds tend to consume more sugar to provide energy for defense substances like PR proteins in order to defend against pathogens.

It is well known that the expression of PR proteins (i.e., chitinases and β-1, 3-glucanases) is often induced as the important aspects of induced systemic resistance during and/or after pathogen attack (Durrant and Dong, 2004; Jain and Khurana, 2018). Chitinases are the enzymes involved in the breaking of the β-1,4-glycosidic linkages of chitin, and they are present in the cotyledons and seed coats of soybean acting as an important seed defense molecule targeting the pathogen cell wall (Gijzen et al., 2001). Yang et al. (2020) reported that the overexpression of the chitinase gene *CmCH1* enhanced soybean resistance to *Sclerotinia sclerotiorum.* β-1, 3-glucanases also belong to the PR-2 family of PR proteins, and they often defend plants against fungal pathogens by degrading the fungal cell wall either alone or in association with chitinase (Balasubramanian et al., 2012). In our study, both chitinase and β-1, 3 glucanase were rapidly activated in soybean seeds at the early infection of *F. fujikuroi*, which might contribute for early seed coat resistance to *F. fujikuroi* infection. To consolidate the pathogenicity, some pathogens have evolved to protein inhibitors to directly inhibit the activities of PRs proteins, such as β-1, 3-glucanases (Rose et al., 2002; Naumann et al., 2009; Sánchez-Rangel et al., 2012). This can explain that the activities of β-1, 3-glucanases gradually decreased after 2 days of *F. fujikuroi* inoculation. Thus, PRs proteins, typically chitinase and β-1, 3-glucanases, can be induced in soybean seeds to defend against *F. fujikuroi* infection.

Plants often need to reprogram their primary metabolism to provide energy and build defenses to cope with adverse environmental stresses (Bolton, 2009; Rojas et al., 2014). In this study, the amino acid metabolism was strongly highlighted among the major metabolic pathways reprogrammed upon *F. fujikuroi* infection (**Supplementary Table S2**). Many studies have demonstrated that the mobilized amino acid pool provides precursors and even energy for the synthesis of a large number of metabolites that are associated with a response to diverse environment stimuli (Hofmann et al., 2010; Deng et al., 2022). Thus, the activation of the amino acid metabolism might contribute to precursors and energy for other metabolites to adapt or defend against *F. fujikuroi*. In addition, we found that the glycine, serine, and threonine metabolism, as well as tryptophan metabolism, were the most metabolic pathways related to *F. fujikuroi* infection at 3 dpi. Previous studies have shown that tryptophan has an important role in plant defense (Servillo et al., 2013; Tunsagool et al., 2019) and is a precursor of serotonin (Ishihara et al., 2008). In this study, L-tryptophan was found to be downregulated at 3 dpi, presumably synthesizing auxins, secondary metabolites which contributing to plant defense (i.e. serotonin). As for tryptophan-derived secondary metabolites with defense, further studies were needed.

Soybean isoflavones are an important class of secondary metabolites and play the vital role in soybean defense against biotic and abiotic stimuli (Chennupati et al., 2012; Qin et al., 2016; Qin et al., 2017). For example, isoflavone accumulation enhanced soybean resistance to the mosaic virus (Hao et al., 2007), cyst nematode (Lin et al., 2016), and field mold (Deng et al., 2017). In our study, the inoculation of *F. fujikuroi* remarkably affected the metabolic profile of soybean seeds and significantly activated the isoflavone pathways and related metabolites. Meanwhile, as the downstream of naringenin and liquiritigenin in the flavonoid pathway, several key metabolites such as genistein, glycitein, daidzein, and genistein were detected to change in different ways upon *F. fujikuroi* infection. Previous studies demonstrated that naringenin is a potential biomarker of resistance to *Fusarium* head blight in some wheat and barley varieties (Gunnaiah et al., 2012) and it has also been used as a potent inhibitor of *F. graminearum in vitro* (Bollina et al., 2010). Deng et al. (2022) showed a significant enrichment of two isoflavone aglycones, genistein and daidzein, in soybean seeds in response to field mold. Morkunas et al. (2010) reported that genistein and genistin were induced to be upregulated at 48 hpi and significantly decreased at 72 hpi using non-inoculated yellow lupine seeds as control, indicating that the two metabolites function at an early infection stage of *F. oxysporum* shortly after the embryonic axis breaks through the seed coat. However, in our study, regarding the seed disease symptoms, isoflavones were not detected at much earlier stage before 3 days of pathogen inoculation; for example, glycitein was only detected to sharply accumulate at 3 dpi to promote soybean seed resistance to *F. fujikuroi*. Meanwhile, in our study, we only compared the metabolite difference of soybean seeds among the different inoculated times of *F. fujikuroi* but did not employ the non-inoculated seeds as control; thus, certain different metabolites might be seed germination and development. Therefore, in further work, the different metabolites upon *F. fujikuroi* inoculation over time need to be verified by high performance liquid chromatography (HPLC) analysis or the gene expression related to metabolite biosynthesis and regulation.

The plant hormone signaling molecules such as jasmonic acid (JA), ehylene (ET), and abscisic acid (ABA), are usually associated with the defense mechanism of plants against necrotrophic pathogens (Glazebrook, 2005; Spoel et al., 2007; Denancé et al., 2013), like the pathogen *F. fujikuroi* (Matić et al., 2016). In our study, the plant hormone signal transduction pathway was significantly enriched in the scatter plots of the top 20 KEGG pathway at 3 dpi compared to control (S88_3.vs.S88_0). Moreover, Hormonal signaling molecules such as JA, ABA, and auxin, were all enriched, implying a crosstalk of plant hormone signaling is activated to defend against *F. fujikuroi* (**Supplementary Figure S1**). Previous studies demonstrated that auxin can act as positive or negative regulators of plant defense (Chen et al., 2007; Llorente et al., 2008; Denancé et al., 2013). In the present study, IAA (Indole-3-acetic acid) was transiently upregulated at 3 dpi but sequentially downregulated, which may be employed by *F. fujikuroi* at early infection stage but latterly repressed to increase soybean susceptiblity to *F. fujikuroi*. In addition, ABA often functions on seed germination and water stress management related to stomata closure in the epidermis (Ali and Baloch, 2020), but it also has different roles at different stages of pathogen infection, in particular, favoring resistance before invasion (Melotto et al., 2006) and susceptibility at later stages of colonization (López et al., 2008). In our study, compared with 0 dpi, ABA was only remarkably upregulated at 3 dpi, probably triggering the stomata closure of inoculated seeds to prevent *F. fujikuroi* penetration. In another study, Siewers et al. (2004) reported that ABA played a negative role in plant resistance to *B. cinerea* when interacted with other hormone signaling molecules such as JA or ET. *B. cinerea*. In this study, JA signaling was clearly downregulated at 3 dpi; however, whether it is related to upregulated ABA signaling needs to be further elucidated in the following work. Overall, *F. fujikuroi* activated a complicated plant hormone signaling net of soybean seeds, and the crosstalk of these hormones might contribute to seed resistance.

## Conclusions

This study, for the first time, demonstrated the physiological and metabolic responses of soybean seeds contributing to its resistance to *F. fujikuroi* in an integrated and complicated way. The seed-borne *F. fujikuroi* severely affected the germination of soybean seeds and changed the contents of soluble sugar and soluble protein in a different way. In addition, *F. fujikuroi* also transiently activated two cell wall–degrading enzymes, chitinase and β-1,3-glucanase, at the early infection stage. A large number of metabolites in glycine, serine, and threonine metabolism and tryptophan metabolism were significantly accumulated, while the metabolites involved in isoflavone biosynthesis and flavonoid biosynthesis were differently induced upon *F. fujikuroi* infection over time. These results are helpful to expand the understanding of the interaction mechanism of soybean and seed-borne pathogens as well as the resistance breeding of soybean.

## Data availability statement

The original contributions presented in the study are included in the article/**Supplementary Material**. Further inquiries can be directed to the corresponding author.

## Author contributions

XC, XL, and HL designed the research. XL, HM, and HL performed the research. XW, HC, and XL analyzed the data. XC and XL wrote the paper. CY, GG, and TL edited the paper. MZ, WC, and WY supervised and funded the research. All authors contributed to the article and approved the submitted version.

## Funding

This study was funded by the Science and Technology Plan of Sichuan Province-Key research and development project (2021YFYZ0018), the National Natural Science Foundation of China (31801685) and Agricultural Science and Technology Innovation Program (CAAS-ASTIP).

## Acknowledgments

The authors thank We thank Mr. Minrong Zhang from Nanchong Agricultural Academical Institution in Sichuan Province, China to provide soybean seeds for our experiments.

## Conflict of interest

The authors declare that the research was conducted in the absence of any commercial or financial relationships that could be construed as a potential conflict of interest.

## Publisher’s note

All claims expressed in this article are solely those of the authors and do not necessarily represent those of their affiliated organizations, or those of the publisher, the editors and the reviewers. Any product that may be evaluated in this article, or claim that may be made by its manufacturer, is not guaranteed or endorsed by the publisher.

## Supplementary material

Supplementary Figure S1 Plant hormone signal transduction pathway at 3 dpi.

Supplementary Table S1 KEGG Pathway enrichment results of differential metabolites at three comparison groups over time after *F. fujikuroi*.

Supplementary Table S2 Changes in metabolites of amino acid metabolic pathways at 3 dpi.

## References

[B1] Ali S. Baloch A. M. (2020). Overview of sustainable plant growth and differentiation and the role of hormones in controlling growth and development of plants under various stresses. Recent Pat. Food Nutr. Agric. 11, 105–114. doi: 10.2174/2212798410666190619104712 31215383

[B2] Balasubramanian V. Vashisht D. Cletus J. Sakthivel N. (2012). Plant β-1, 3-glucanases: their biological functions and transgenic expression against phytopathogenic fungi. Biotechnol. Lett. 34, 1983–1990. doi: 10.1007/s10529-012-1012-6 22850791

[B3] Bollina V. Kumaraswamy G. K. Kushalappa A. C. Choo T. M. Dion Y. Rioux S. . (2010). Mass spectrometry-based metabolomics application to identify quantitative resistance-related metabolites in barley against *Fusarium* head blight. Mol. Plant Pathol. 11, 769–782. doi: 10.1111/j.1364-3703.2010.00643.x 21029322 PMC6640360

[B4] Bolton M. D. (2009). Primary metabolism and plant defense–fuel for the fire. Mol. Plant Microbe Interact. 22, 487–497. doi: 10.1094/MPMI-22-5-0487 19348567

[B5] Cen Y. K. Lin J. G. Wang Y. L. Wang J. Y. Liu Z. Q. Zheng Y. G. (2020). The gibberellin producer *Fusarium fujikuroi*: Methods and technologies in the current toolkit. Front. Bioeng Biotech. 8. doi: 10.3389/fbioe.2020.00232 PMC7118215 32292777

[B6] Chang X. L. Li H. J. Naeem M. Wu X. L. Yong T. W. Song C. . (2020a). Diversity of the seedborne fungi and pathogenicity of *Fusarium* species associated with intercropped soybean. Pathogens. 9, 531. doi: 10.3390/pathogens9070531 PMC7400112 32630289

[B7] Chang X. L. Naeem M. Li H. J. Yan L. Liu T. G. Liu B. . (2020b). First report of *Fusarium asiaticum* as a causal agent for seed decay of soybean (*Glycine max*) in sichuan, China. Plant Dis. 104, 1542–1542. doi: 10.1094/PDIS-11-19-2488-PDN

[B8] Chen L. Wu Q. He W. He T. Wu Q. Miao Y. (2019). Combined *de novo* transcriptome and metabolome analysis of common bean response to *Fusarium oxysporum* f. sp. *phaseoli* infection. Int. J. Mol. Sci. 20, 6278. doi: 10.3390/ijms20246278 PMC6941151 31842411

[B9] Chen Z. Agnew J. L. Cohen J. D. He P. Shan L. Sheen J. . (2007). Pseudomonas syringae type III effector AvrRpt2 alters *Arabidopsis thaliana* auxin physiology. Proc. Natl. Acad. Sci. U S A. 104, 20131–20136. doi: 10.1073/pnas.0704901104 18056646 PMC2148434

[B10] Chen X. Y. Kim J. Y. (2009). Callose synthesis in higher plants. Plant Signal Behav. 4, 489–492. doi: 10.4161/psb.4.6.8359 19816126 PMC2688293

[B11] Chennupati P. Seguin P. Chamoun R. Jabaji S. (2012). Effects of high-temperature stress on soybean isoflavone concentration and expression of key genes involved in isoflavone synthesis. J. Agric. Food Chem. 60, 12421–12427. doi: 10.1021/jf3036319 23199070

[B12] Chiotta M. L. Alaniz Zanon M. S. Palazzini J. M. Scandiani M. M. Formento A. N. Barros G. G. . (2016). Pathogenicity of *Fusarium graminearum* and *F. meridionale* on soybean pod blight and trichothecene accumulation. Plant Pathol. 65, 1492–1497. doi: 10.1111/ppa.12532

[B13] Denancé N. Sánchez-Vallet A. Goffner D. Molina A. (2013). Disease resistance or growth: the role of plant hormones in balancing immune responses and fitness costs. Front. Plant Sci. 4. doi: 10.3389/fpls.2013.00155 PMC3662895 23745126

[B14] Deng J. C. Li X. M. Xiao X. L. Wu H. J. Yang C. Q. Long X. Y. . (2022). Field mold stress induced catabolism of storage reserves in soybean seed and the resulting deterioration of seed quality in the field. J. Integr. Agr. 21, 336–350. doi: 10.1016/S2095-3119(20)63594-8

[B15] Deng J. C. Yang C. Q. Zhang J. Zhang Q. Yang F. Yang W. Y. . (2017). Organ-specifific differential NMR-based metabonomic analysis of soybean [*Glycine max* (L.) merr.] rruit reveals the metabolic shifts and potential protection mechanisms involved in field mold infection. Front. Plant Sci. 8. doi: 10.3389/fpls.2017.00508 PMC5404178 28487702

[B16] Dodds P. N. Rathjen J. P. (2010). Plant immunity: towards an integrated view of plant-pathogen interaction. Nat. Rev. Genet. 11, 539–548. doi: 10.1038/nrg2812 20585331

[B17] Durrant W. E. Dong X. (2004). Systemic acquired resistance. Annu. Rev. Phytopathol. 42, 185–209. doi: 10.1146/annurev.phyto.42.040803.140421 15283665

[B18] Egea C. Alcázar M. D. Candela M. E. (1996). β-1,3-glucanase and chitinase as pathogenesis-related proteins in the defense reaction of two *Capsicum annuum* cultivars infected with cucumber mosaic virus. Biol. Plantarum. 38, 437–443. doi: 10.1007/BF02896676

[B19] Formela-Luboińska M. Chadzinikolau T. Drzewiecka K. Jeleń H. Bocianowski J. Kęsy J. . (2020). The role of sugars in the regulation of the level of endogenous signaling molecules during defense response of yellow lupine to *Fusarium oxysporum* . Int. J. Mol. Sci. 21, 4133. doi: 10.3390/ijms21114133 PMC7312090 32531938

[B20] Gijzen M. Kuflu K. Qutob D. Chernys J. T. (2001). A class I chitinase from soybean seed coat. J. Exp. Bot. 52, 223–2289. doi: 10.1093/jexbot/52.365.2283 11709578

[B21] Glazebrook J. (2005). Contrasting mechanisms of defense against biotrophic and necrotrophic pathogens. Annu. Rev. Phytopathol. 43, 205–227. doi: 10.1146/annurev.phyto.43.040204.135923 16078883

[B22] González-Lamothe R. Mitchell G. Gattuso M. Diarra M. S. Malouin F. Bouarab K. (2009). Plant antimicrobial agents and their effects on plant and human pathogens. Int. J. Mol. Sci. 10, 3400–3419. doi: 10.3390/ijms10083400 20111686 PMC2812829

[B23] Gunnaiah R. Kushalappa A. C. Duggavathi R. Fox S. Somers D. J. (2012). Integrated metabolo-proteomic approach to decipher the mechanisms by which wheat QTL (*Fhb1*) contributes to resistance against *Fusarium graminearum* . PLoS One 7, e40695. doi: 10.1371/journal.pone.0040695 22866179 PMC3398977

[B24] Hao J. Ma H. Q. Dai R. Chen S. W. (2007). Construction and expression of the soybean isoflavonoid biosynthetic pathway in *Escherichia coli* . Chin. J. Biotechnol. 23, 1022–1028. doi: 10.13345/j.cjb.2007.06.001 18257230

[B25] Hobbs T. W. Schmitthenner A. F. Kuter G. A. (1985). A new phomopsis species from soybean. Mycologia. 77, 535–544. doi: 10.1080/00275514.1985.12025139

[B26] Hofmann J. El Ashry A. E. N. Anwar S. Erban A. Kopka J. Grundler F. (2010). Metabolic profiling reveals local and systemic responses of host plants to nematode parasitism. Plant J. 62, 1058–1071. doi: 10.1111/j.1365-313X.2010.04217.x 20374527 PMC2904900

[B27] Ishihara A. Hashimoto Y. Tanaka C. Dubouzet J. G. Nakao T. Matsuda F. . (2008). The tryptophan pathway is involved in the defense responses of rice against pathogenic infection *via* serotonin production. Plant J. 54, 481–495. doi: 10.1111/j.1365-313X.2008.03441.x 18266919

[B28] Jain D. Khurana J. P. (2018). “Role of pathogenesis-related (PR) proteins in plant defense mechanism,” in Molecular aspects of plant-pathogen interaction (Singapore: Springer). doi: 10.1007/978-981-10-7371-7_12

[B29] Jiang H. Wu N. Jin S. Ahmed T. Wang H. Li B. . (2020). Identification of rice seed-derived fusarium spp. and development of LAMP assay against *Fusarium fujikuroi* . Pathogens. 10, 1. doi: 10.3390/pathogens10010001 33374990 PMC7822049

[B30] Jung W. Yu O. Lau S. M. C. O'Keefe D. P. Odell J. Fader G. . (2000). Identification and expression of isoflavone synthase, the key enzyme for biosynthesis of isoflavones in legumes. Nat. Biotechnol. 18, 208–212. doi: 10.1038/72671 10657130

[B31] Leslie J. F. Zeller K. A. Lamprecht S. C. Rheeder J. P. Marasas W. F. O. (2005). Toxicity, pathogenicity, and genetic differentiation of five species of *Fusarium* from sorghum and millet. Phytopathology. 95, 275–283. doi: 10.1094/PHYTO-95-0275 18943121

[B32] Li H. S. (2002). Modern plant physiology (Beijing: Higher Education Press).

[B33] Liang X. Q. Pan. R. Z. Bin J. H. (2000). Progress of research on the mechanism of resistance to *Aspergillus flavus* infestation in peanuts. Chin. J. Oil Crop Sci. 22, 77–80. doi: 10.3321/j.issn:1007-9084.2000.03.020

[B34] Li S. Hartman G. L. Boykin D. L. (2010). Aggressiveness of *Phomopsis longicolla* and other *Phomopsis* spp. on soybean. Plant Dis. 94, 1035–1040. doi: 10.1094/PDIS-94-8-1035 30743477

[B35] Lin X. M. Tan X. R. Wang L. Z. Li B. Sun J. M. (2016). Root isoflavone content after cyst nematode infection in different soybean varieties. Chin. J. Oil Crop Sci. 38, 495. doi: 10.7505/j.issn.1007-9084.2016.04.013

[B36] Li S. Rupe J. Chen P. Shannon G. Wrather A. Boykin D. (2015). Evaluation of diverse soybean germplasm for resistance to *Phomopsis* seed decay. Plant Dis. 99, 1517–1525. doi: 10.1094/PDIS-04-14-0429-RE 30695950

[B37] Li S. Sciumbato G. Boykin D. Shannon G. Chen P. (2019). Evaluation of soybean genotypes for reaction to natural field infection by *Cercospora* species causing purple seed stain. PLoS One 14, e0222673. doi: 10.1371/journal.pone.0222673 31600229 PMC6786595

[B38] Li S. Sciumbato G. Rupe J. Shannon G. Chen P. Boykin D. (2017). Evaluation of commercial soybean cultivars for reaction to *Phomopsis* seed decay. Plant Dis. 101, 1990–1997. doi: 10.1094/PDIS-02-17-0204-RE 30677383

[B39] Li X. M. Yang C. Q. Chen J. H. He Y. Y. Deng J. C (2021). Changing light promotes isoflavone biosynthesis in soybean pods and enhances their resistance to mildew infection. Plant Cell Environ 44, 2536. doi: 10.1111/pce.14150 34118074

[B40] Llorente F. Muskett P. Sanchez-Vallet A. López G. Ramos B. Sanchez-Rodriguez C. . (2008). Repression of the auxin response pathway increases arabidopsis susceptibility to necrotrophic fungi. Mol. Plant 1, 496–509. doi: 10.1093/mp/ssn025 19825556

[B41] López M. A. Bannenberg G. Castresana C. (2008). Controlling hormone signaling is a plant and pathogen challenge for growth and survival. Curr. Opin. Plant Biol. 11, 420–427. doi: 10.1016/j.pbi.2008.05.002 18585953

[B42] Luna E. Pastor V. Robert J. Flors V. Mauch-Mani B. Ton J. (2011). Callose deposition: A multifaceted plant defense response. Mol. Plant Microbe Interact. 24, 183–193. doi: 10.1094/MPMI-07-10-0149 20955078

[B43] Lv G. Z. Sun Y. W. (2007). Soybean pests and diseases and control of the original color atlas (Beijing: Golden Shield Publishing House).

[B44] Maher E. A. Lamb C. J. Dixon R. A. (1993). Stress responses in alfalfa (*Medicago sativa* l) XVII. identification of multiple hydrolases and molecular characterization of an acidic glucanase. Physiol. Mol. Plant P. 43, 329–342. doi: 10.1006/pmpp.1993.1062

[B45] Mao B. Liu X. Hu D. Li D. (2014). Co-Expression of *RCH10* and *AGLU1* confers rice resistance to fungal sheath blight *Rhizoctonia solani* and blast *Magnorpathe oryzae* and reveals impact on seed germination. World J. Microbiol. Biotechnol. 30, 1229–1238. doi: 10.1007/s11274-013-1546-3 24197785

[B46] Matić S. Bagnaresi P. Biselli C. Orru L. Amaral Carneiro G. Siciliano I. . (2016). Comparative transcriptome profiling of resistant and susceptible rice genotypes in response to the seedborne pathogen *Fusarium fujikuroi* . BMC Genomics 17, 1–17. doi: 10.1186/s12864-016-2925-6 27515776 PMC4981969

[B47] Melotto M. Underwood W. Koczan J. Nomura K. He S. Y. (2006). Plant stomata function in innate immunity against bacterial invasion. Cell. 126, 969–980. doi: 10.1016/j.cell.2006.06.054 16959575

[B48] Miedes E. Vanholme R. Boerjan W. Molina A (2014). The role of the secondary cell wall in plant resistance to pathogens. Front. Plant Sc 5, 358. doi: 10.3389/fpls.2014.00358 25161657 PMC4122179

[B49] Morkunas I. Ratajczak L. (2014). The role of sugar signaling in plant defense responses against fungal pathogens. Acta Physiol. Plant 36, 1607–1619. doi: 10.1007/s11738-014-1559-z

[B50] Morkunas I. Stobiecki M. Marczak Ł. Stachowiak J. Narożna D. Remlein-Starosta D. (2010). Changes in carbohydrate and isoflavonoid metabolism in yellow lupine in response to infection by fusarium oxysporum during the stages of seed germination and early seedling growth. Physiol. Mol. Plant P. 75, 46–55. doi: 10.1016/j.pmpp.2010.08.005

[B51] Naumann T. A. Wicklow D. T. Kendra D. F. (2009). Maize seed chitinase is modified by a protein secreted by *Bipolaris zeicola* . Physiol. Mol. Plant P. 74, 134–141. doi: 10.1016/j.pmpp.2009.10.004

[B52] O'Donnell K. Cigelnik E. Nirenberg H. I. (1998). Molecular systematics and phylogeography of the *Gibberella fujikuroi* species complex. Mycologia. 90, 465–493. doi: 10.1080/00275514.1998.12026933

[B53] Petrović K. Riccioni L. Vidić M. Đorđević V. Balešević-Tubić S. Đukić V. . (2016). First report of *Diaporthe novem*, *D. foeniculina*, and *D. rudis* associated with soybean seed decay in Serbia. Plant Dis. 100, 2324–2324. doi: 10.1094/PDIS-03-16-0353-PDN

[B54] Purahong W. Alkadri D. Nipoti P. Pisi A. Lemmens M. Prodi A. (2012). Validation of a modified petri-dish test to quantify aggressiveness of *Fusarium graminearum* in durum wheat. Eur. J. Plant Pathol. 132, 381–391. doi: 10.1007/s10658-011-9883-2

[B55] Qin W. T. Feng Y. R. Lei Z. Yang C. Q. Wu H. J. NASIR I. . (2017). Effect of shading signal on isoflavone biosynthesis of soybean seedling. Natural Product Res. Dev. 29 (9), 1470–1474. doi: 10.16333/j.1001-6880.2017.9.003

[B56] Qin W. T. Zhang J. Wu H. J. Sun G. Z. Yang W. Y. Liu J. (2016). Effects of drought stress on biosynthesis of isoflavones in soybean seedling. J. Appl. Ecology. 27, 3927–3934. doi: 10.13287/j.1001-9332.201612.018 29704352

[B57] Rojas C. M. Senthil-Kumar M. Tzin V. Mysore K. S. (2014). Regulation of primary plant metabolism during plant-pathogen interactions and its contribution to plant defense. Front. Plant Sci. 5. doi: 10.3389/fpls.2014.00017 PMC3919437 24575102

[B58] Rose J. K. Ham K. S. Darvill A. G. Albersheim P. (2002). Molecular cloning and characterization of glucanase inhibitor proteins: coevolution of a counterdefense mechanism by plant pathogens. Plant Cell. 14, 1329–1345. doi: 10.1105/tpc.002253 12084830 PMC150783

[B59] Sánchez-Rangel D. Sánchez-Nieto S. Plasencia J. (2012). Fumonisin B1, a toxin produced by *Fusarium verticillioides*, modulates maize β-1, 3-glucanase activities involved in defense response. Planta. 235, 965–978. doi: 10.1007/s00425-011-1555-0 22120123

[B60] Servillo L. Giovane A. Balestrieri M. L. Casale R. Cautela D. Castaldo D. (2013). Citrus genus plants contain n-methylated tryptamine derivatives and their 5-hydroxylated forms. J. Agric. Food Chem. 61, 5156–5162. doi: 10.1021/jf401448q 23682903

[B61] Shi X. Chen Q. Liu S. Wang J. Peng D. Kong L. (2021). Combining targeted metabolite analyses and transcriptomics to reveal the specific chemical composition and associated genes in the incompatible soybean variety PI437654 infected with soybean cyst nematode HG1. 2.3. 5.7. BMC Plant Biol. 21, 1–17. doi: 10.1186/s12870-021-02998-4 33990182 PMC8120846

[B62] Siewers V. Smedsgaard J. Tudzynski P. (2004). The P450 monooxygenase BcABA1 is essential for abscisic acid biosynthesis in *Botrytis cinerea* . Appl. Environ. Microbiol. 70, 3868–3876. doi: 10.1128/AEM.70.7.3868-3876.2004 15240257 PMC444755

[B63] Soni P. Nayak S. N. Kumar R. Pandey M. K. Singh N. Sudini H. K. . (2020). Transcriptome analysis identified coordinated control of key pathways regulating cellular physiology and metabolism upon *Aspergillus flavus* infection resulting in reduced aflatoxin production in groundnut. J. Fungi (Basel). 6, 370. doi: 10.3390/jof6040370 PMC7767264 33339393

[B64] Spoel S. H. Johnson J. S. Dong X. (2007). Regulation of tradeoffs between plant defenses against pathogens with different lifestyles. Proc. Natl. Acad. Sci. U S A. 104, 18842–18847. doi: 10.1073/pnas.0708139104 17998535 PMC2141864

[B65] Steele C. L. Gijzen M. Qutob D. Dixon R. A. (1999). Molecular characterization of the enzyme catalyzing the aryl migration reaction of isoflavonoid biosynthesis in soybean. Arch. Biochem. Biophys. 367, 146–150. doi: 10.1006/abbi.1999.1238 10375412

[B66] Suga H. Arai M. Fukasawa E. Motohashi K. Nakagawa H. Tateishi H. . (2019). Genetic differentiation associated with fumonisin and gibberellin production in Japanese *Fusarium fujikuroi* . Appl. Environ. Microbiol. 85, e02414–e02418. doi: 10.1128/AEM.02414-18 30341078 PMC6293107

[B67] Summerell B. A. Laurence M. H. Liew E. C. Y. Leslie J. F. (2010). Biogeography and phylogeography of *Fusarium*: a review. Fungal Divers. 44, 3–13. doi: 10.1080/19440049.2014.984244

[B68] Sun S. K. Snyder. W. C. (1981). “The bakanae disease of the rice plant,” in Fusarium: Diseases, biology, and taxonomy (University Park, Pennsylvania: Pennsylvania State Univ.), 104–113.

[B69] Tarkowski Ł.P. Van de Poel B. Höfte M. Van den Ende W. (2019). Sweet immunity: Inulin boosts resistance of lettuce (*Lactuca sativa*) against grey mold (*Botrytis cinerea*) in an ethylene-dependent manner. Int. J. Mol. Sci. 20, 1052. doi: 10.3390/ijms20051052 PMC6429215 30823420

[B70] Torres M. A. Jones J. D. Dangl J. L. (2006). Reactive oxygen species signaling in response to pathogens. Plant Physiol. 141, 373–378. doi: 10.1104/pp.106.079467 16760490 PMC1475467

[B71] Tunsagool P. Wang X. Leelasuphakul W. Jutidamrongphan W. Phaonakrop N. Jaresitthikunchai J. . (2019). Metabolomic study of stress responses leading to plant resistance in mandarin fruit mediated by preventive applications of *Bacillus subtilis* cyclic lipopeptides. Postharvest Biol. Tec. 156, 110946. doi: 10.1016/j.postharvbio.2019.110946

[B72] Wrather A. Koenning S. (2009). Effects of diseases on soybean yields in the united states 1996 to 2007. Plant Health Prog. 10, 24. doi: 10.1094/PHP-2009-0401-01-RS

[B73] Wrather J. A. Sleper D. A. Stevens W. E. Shannon J. G. Wilson R. F. (2003). Planting date and cultivar effects on soybean yield, seed quality, and *Phomopsis* sp. seed infection. Plant Dis. 87, 529–532. doi: 10.1094/PDIS.2003.87.5.529 30812953

[B74] Wu Y. B. Xie L. Y. Xie L. H. Lin Q. Y. (2007). Effects of coprinus comatus polysaccharide on the activities of enzymes and isoenzyme zymogram in tobacco leaves. J. Microbiol. 05, 29–33. doi: 10.3969/j.issn.1005-7021.2007.05.007

[B75] Xiong Q. E. (2003). Plant physiology laboratory tutorial (Chengdu: Sichuan Science and Technology Press).

[B76] Yang X. Yang J. Li H. Niu L. Xing G. Zhang Y. . (2020). Overexpression of the chitinase gene *CmCH1* from *Coniothyrium minitans* renders enhanced resistance to *Sclerotinia sclerotiorum* in soybean. Transgenic Res. 29, 187–198. doi: 10.1007/s11248-020-00190-2 31970612

[B77] Yeoh K. A. Othman A. Meon S. Abdullah F. Ho C. L. (2012). Sequence analysis and gene expression of putative exo-and endo-glucanases from oil palm (*Elaeis guineensis*) during fungal infection. J. Plant Physiol. 169, 1565–1570. doi: 10.1016/j.jplph.2012.07.006 22854183

[B78] Zhang A. W. Riccioni L. Pedersen W. L. Kollipara K. P. Hartman G. L. (1998). Molecular identification and phylogenetic grouping of *Diaporthe phaseolorum* and *Phomopsis longicolla* isolates from soybean. Phytopathology. 88, 1306–1314. doi: 10.1094/PHYTO.1998.88.12.130 18944833

[B79] Zhou Y. Zhu Y. Li Y. Duan Y. Zhang R. Zhou M. (2016). β1 tubulin rather than β2 tubulin is the preferred binding target for carbendazim in *Fusarium graminearum* . Phytopathology. 106, 978–985. doi: 10.1094/PHYTO-09-15-0235-R 27135676

