# Supplementary material for: Physiological and metabolic analyses provide insight into soybean seed resistance to fusarium fujikuroi causing seed decay
Source: Front Plant Sci. 2022 Oct 20;13:993519. doi: 10.3389/fpls.2022.993519 (PMC9630849; doi:10.3389/fpls.2022.993519)
Supplement: Supplementary Figure S1 — Plant hormone signal transduction pathway at 3 dpi. [file DataSheet_1.docx]

**Supplementary Materials**


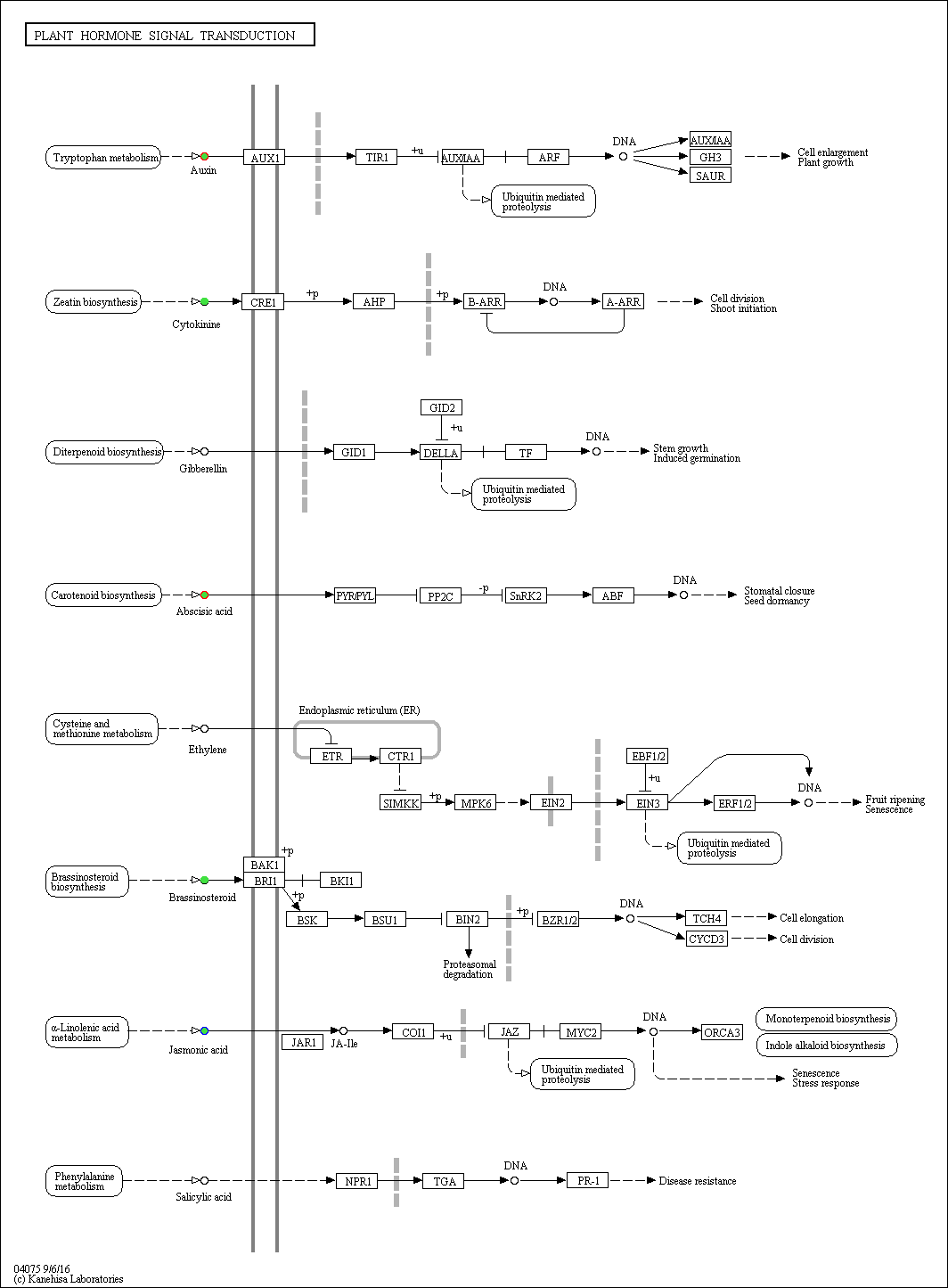


**Supplementary Figure S1 Plant hormone signal transduction pathway at 3 dpi.** In the KEGG pathway diagram, circles represent metabolites, where green solid circles mark metabolites annotated to, red circles mark up-regulated differential metabolites, blue circles mark down-regulated differential metabolites, and yellow circles indicate metabolites containing both up- and down-regulated metabolites.
